# Anterior Medial Prefrontal Cortex Exhibits Activation during Task Preparation but Deactivation during Task Execution

**DOI:** 10.1371/journal.pone.0022909

**Published:** 2011-08-01

**Authors:** Hideya Koshino, Takehiro Minamoto, Takashi Ikeda, Mariko Osaka, Yuki Otsuka, Naoyuki Osaka

**Affiliations:** 1 Department of Psychology, California State University, San Bernardino, California, United States of America; 2 Department of Psychology, Kyoto University, Kyoto, Japan; 3 Department of Human Sciences, Osaka University, Osaka, Japan; The University of Melbourne, Australia

## Abstract

**Background:**

The anterior prefrontal cortex (PFC) exhibits activation during some cognitive tasks, including episodic memory, reasoning, attention, multitasking, task sets, decision making, mentalizing, and processing of self-referenced information. However, the medial part of anterior PFC is part of the default mode network (DMN), which shows deactivation during various goal-directed cognitive tasks compared to a resting baseline. One possible factor for this pattern is that activity in the anterior medial PFC (MPFC) is affected by dynamic allocation of attentional resources depending on task demands. We investigated this possibility using an event related fMRI with a face working memory task.

**Methodology/Principal Findings:**

Sixteen students participated in a single fMRI session. They were asked to form a task set to remember the faces (Face memory condition) or to ignore them (No face memory condition), then they were given 6 seconds of preparation period before the onset of the face stimuli. During this 6-second period, four single digits were presented one at a time at the center of the display, and participants were asked to add them and to remember the final answer. When participants formed a task set to remember faces, the anterior MPFC exhibited activation during a task preparation period but deactivation during a task execution period within a single trial.

**Conclusions/Significance:**

The results suggest that the anterior MPFC plays a role in task set formation but is not involved in execution of the face working memory task. Therefore, when attentional resources are allocated to other brain regions during task execution, the anterior MPFC shows deactivation. The results suggest that activation and deactivation in the anterior MPFC are affected by dynamic allocation of processing resources across different phases of processing.

## Introduction

The anterior PFC (BA 10, also called frontal pole, frontpolar cortex, and rostral prefrontal cortex) exhibits activation in various higher-level cognitive tasks, including episodic memory retrieval [1]–[8], prospective memory [9]–[11], working memory [12], attention [13]–[16], reasoning [17], [18], multitasking [19]–[23], rule learning [24]–[27], internally generated information [28], [29], monitoring of external environment [30], formation and management of task sets and rules [31]–[35], decision making [36], [37], and processing of self-referenced information [38], [39].

There might be some functional subdivisions within the BA10 [40]–[42]. For example, Gilbert et al. [42] reported that functions such as episodic memory are more related to the lateral BA10, whereas mentalizing is associated with the medial BA10, even though some functions such as attention and multitasking are related to both the lateral and medial BA10. It has also been reported that patients with damage to the anterior PFC do not seem to show deficits in processing of some of the functions, except that they showed a significant impairment in multitasking [43]. Based on this observation, Burgess et al. [30] suggested that it seems that some regions of the anterior PFC are more involved with the intention to perform a task rather than with task execution.

The medial part of anterior PFC is part of the default mode network (DMN) and has been known to show task induced deactivation (TID) during some cognitive tasks compared to a resting baseline [44]–[51]. The DMN also includes the posterior cingulate/precuneus (PCC), inferior parietal lobe (IPL), lateral temporal cortex (LTC), and hippocampal formation (HF) [45]. There have been debates over mechanisms of the DMN. Some researchers suggested that the DMN is related to internal mentation and mental simulation such as internally focused thought [46], [47] and mind wandering [52], [53]. Other researchers suggested that the DMN is associated with monitoring of the external environment [30], [50]. Also, negative correlations were found between the DMN and other brain regions including the executive network. Specifically, the higher the activation in the executive network, the lower the activation in the DMN [52], [54]–[58]. Mayer et al. [59] also reported that deactivation in the DMN is related to task demands. In their recent review, Buckner et al. [45] suggested that the DMN and the external attention system are competing with each other. The DMN tends to reduce activation when attention is focused on a particular task, whereas the DMN tends to increase activation when attention is rather relaxed. When combined together, these findings suggest that the level of activity in the anterior MPFC may depend on allocation of attentional resources among brain regions as a function of task demands.

Previous studies in TID have also shown that allocation of attention could modulate activation of the task relevant regions [60]–[65]. For example, in the posterior visual areas, the regions corresponding to attended stimuli showed activation, whereas the regions corresponding to unattended stimuli showed deactivation [66]–[69]. Task demand is also related to activation and deactivation [70]–[72]. One potential mechanism of this type of TID involves changes in neural activity due to dynamic allocation of attention [49], [69], [73]. When attention is focused on particular stimuli or spatial locations, the neural activities of the relevant brain regions increase, but those of irrelevant regions decrease, resulting in deactivation.

These studies have investigated the effects of attention on different regions in the brain; however, it also seems possible that allocation of attentional resources has different effects on different temporal phases of information processing. A particular region might show dynamic changes in activation as task demands change across different phases. If this is the case, then it is possible that activation and deactivation in the anterior MPFC are modulated by allocation of attentional resources among different brain regions depending on task demands. In order to investigate this possibility, we manipulated two phases of processing: task preparation and task execution within a single trial. If the anterior MPFC is related to task set formation, it should show activation during the preparation period. However, if the anterior MPFC is not involved in task execution, it should show deactivation during the execution period as the demands on attentional resources in the other brain regions increase.

## Methods

### Participants

Sixteen students from the Kyoto area, (age ranges from 20 to 31 years; 8 females, all right-handers) participated as paid volunteers. All had normal or corrected to normal vision. They gave written informed consent to participate in the study which was approved by the institutional review board of the Advanced Telecommunications Research Institute International (ATR).

### Stimuli

The primary task was face recognition in which participants were required to remember three faces arranged in a triangular or reverse triangular fashion around the center of the screen (visual angle from the center of the screen to the center of the face was 2.3°). The stimulus faces were 168 images of Caucasian men and women retrieved from the Productive Aging Laboratory [74], University of Texas at Dallas (http://agingmind.cns.uiuc.edu/facedb/) and the Psychological Image Collection at Stirling, Psychology Department, University of Stirling (http://pics.psych.stir.ac.uk/). These images were converted to grayscale with the hair and ears trimmed. The visual angle of each face was 2.7°×2.7°. A background task was simple addition of four single-digits presented successively one at a time at the center of the screen. The size of each digit was 0.6°.

### Procedure

A trial began with an auditory instruction to form a task set to remember faces (Face memory condition) or to ignore them (No face memory condition). In the Face memory condition, the participants were asked to form a task set to remember the faces during the preparation period, and in the No face memory condition, they were asked to ignore the faces and therefore no task set was required.

**Figure 1:**
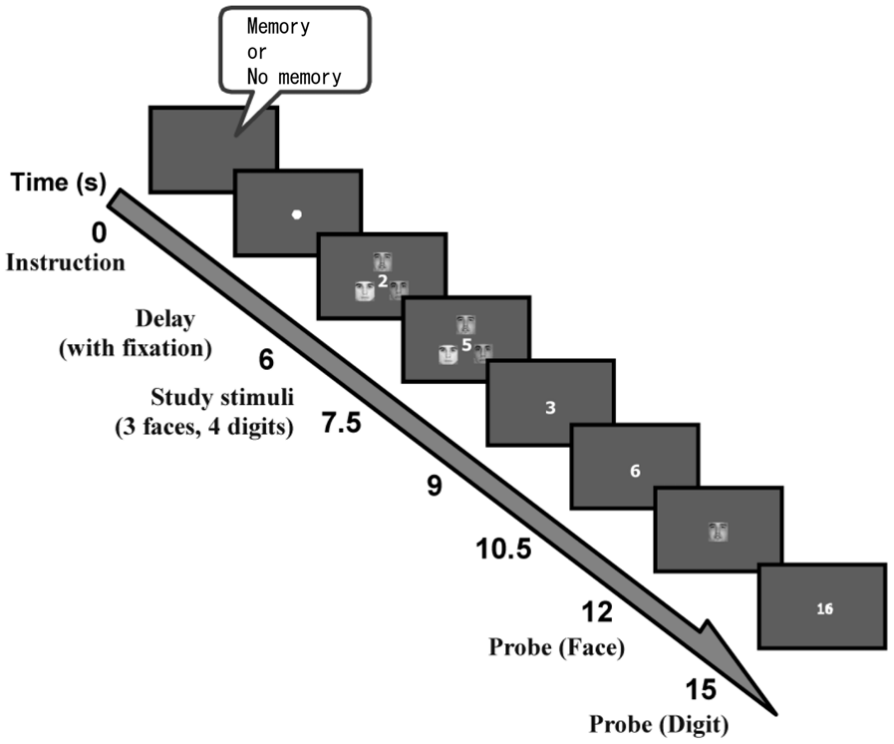
An example of trial sequence. At the beginning of each trial, an auditory instruction was given regarding the formation of task sets (Memory task set or No memory task set condition), followed by a 6-sec delay (Preparation period). Then the task execution period began with stimulus faces presented for 3-sec, followed by a 3-sec delay. During this 6-sec period, four single-digits were presented one at a time for 1.5-sec each, and the participants were asked to add them and remember the final answer. After the delay, a probe face was presented for 1-sec and the participants were given 3-sec to judge whether or not the probe face was among the three faces for the Memory task set condition. For the No memory task set condition, they did not have to respond to the faces. Then a two-digit number was presented for 1-sec and they were asked to decide whether or not the number was the answer to the addition.

Then participants were given 6 seconds of preparation period before the onset of the face stimuli. The task execution period started with a stimulus display consisting of three faces for 3 seconds, then a 3-second delay. During this 6-second period, four single digits were presented one at a time for 1.5 seconds each at the center of the display, and participants were asked to add them and to remember the final answer. This calculation task was added to prevent the use of verbal strategy. Then a test face was presented at the center for 3 seconds, and the participants made a match - no match judgment in the Face memory condition by pressing a response button with the right index finger for match, and with right middle finger for non-match. The proportions of matches and non-matches were 50–50, and no face stimulus was repeated. A response was not required for the No face memory condition. Then, a two-digit number was presented for 3 seconds in both conditions, and participants judged whether or not this number was the correct answer to the addition by pressing a response button with the right index finger for “Yes”, and with right middle finger for “No”. The inter-trial interval (ITI) was varied among 6, 8, or 10 seconds in order to minimize effects of the rest period. An example of trial sequence is shown in Figure 1.

Stimuli were projected onto a viewing screen attached within the bore of the scanner. Stimulus presentation and behavioral data collection were controlled with the Presentation software (Neurobehavioral Systems Inc., Albany, CA, USA). Each participant received a separate practice session before the MRI session. There were 20 trials for each condition, presented in a random order.

### fMRI acquisition

Event-related functional magnetic resonance imaging (fMRI) data were acquired on a 1.5-T whole-body magnetic resonance imaging scanner (Shimadzu-Marconi Magnex Eclipse, Kyoto, Japan). For functional imaging, a gradient-echo echo-planer imaging sequence was used with the following parameters: TR = 2000 ms, TE = 48 ms, flip angle = 80°, and 20 oblique axial slices were taken with 7 mm slice thickness, FOV = 224 mm×224 mm, and pixel matrix = 64×64, with 3.5×3.5×7 mm voxels. Then, T1-weighted images (191 slices with no gap), using a conventional spin-echo pulse sequence (TR = 12 ms, TE = 4.5 ms, flip angle = 20°, FOV = 256 mm×256 mm, and pixel matrix = 256×256, with voxel size 1×1×1 mm), were collected for anatomical co-registration.

Functional images were analyzed using SPM2 (Wellcome Department of Cognitive Neurology, University College London, UK). Six initial images were discarded to eliminate nonequilibrium effects of magnetization. Preprocessing included slice-time correction, motion correction, normalization to the Montreal Neurological Institute (MNI) EPI template, resampled to 2×2×2 mm voxels, and spatially smoothed (Gaussian kernel, full-width at half maximum = 8 mm). In a statistical model, we included separate covariates for the instruction of each condition (preparation period), one for the presentation of visual stimuli (execution period), and one for the inter-trial interval, and convolved those covariates with a hemodynamic response function (HRF), following the procedure employed in our previous study [75]. Event duration of each covariate was 0. No significant correlation was observed among regressors between the preparation period and execution period in each condition, indicating no collinearity among regressors. An uncorrected height threshold (*p* = 0.001) and an extent threshold (10 voxels) were used.

### Regions of Interests (ROI)

We also performed a timecourse analysis of ROIs that are chosen based on the voxelwise analysis, in which activation and deactivation are identified as differences between the Face memory and No Face memory conditions. However, in the timecourse analysis, activation and deactivation in each ROI are determined in comparison with its own baseline for Face memory and No Face memory conditions separately. We expected that these two types of analyses would provide converging evidence.

Sixteen functional ROIs were defined based on the results of the voxelwise analysis. We focused on the regions that were identified in the comparison between Face Memory and No Face Memory during the preparation and execution periods, as shown in Table 1. A sphere was created for each cluster of activation with variable size (Mean radius = 3.8 mm, range = 2 to 6 mm) to maximize the coverage of activation of individual participants [76]. The functional ROIs were all bilateral, including the frontal pole (FP, BA10), orbitofrontal cortex (OFC, BA11), rostral anterior cingulate cortex (ACCr, BA32), caudal anterior cingulate cortex (ACCc, BA32), lateral PFC (LPFC, BA45), intraparietal sulcus (IPS, BA7), inferior temporal lobe (IT, BA37), and inferior extrastriate cortex (IES, BA19). Then the activation time course for each ROI was extracted separately for each participant for each condition using the MarsBaR [77]. A percent signal change (psc) was computed for each ROI with the time point 0 sec as the reference point in order to examine activation and deactivation compared to its own baseline. A 99% confidence interval was computed for each data point to examine whether or not each data point is different from the baseline (psc = 0).

**Table 1:** Areas of activation for the preparation and execution periods between the Face memory and No face memory conditions.

				Talairach Coordinates			
Region	L/R	BA	Cluster Size	x	y	z	T-score
**Preparation:** **Face memory > No face memory**							
Frontal Pole	L	10	71	−12	55	5	5.99
Orbitofrontal Cortex	R	11	32	24	21	−13	5.49
Middle Frontal Gyrus	L	6	26	−22	12	38	5.01
Anterior Cingulate Cortex	L	32	10	−8	37	7	4.27
Inferior Extrastriate Cortex	L	17	11	−22	−87	1	4.60
Inferior Extrastriate Cortex	L	18	70	−30	−76	−8	4.85
**Preparation:** **No face memory > Face memory** **N/A**							
**Execution:** **Face memory > No face memory**							
Lateral Prefrontal Cortex	L	45	817	−40	28	12	8.05
Insula	L	47		−32	25	−1	4.75
Putamam	L	—		−18	7	−5	6.17
Lateral Prefrontal Cortex	L	44	352	−40	5	31	6.67
Lateral Prefrontal Cortex	R	44	1456	42	13	20	6.46
Insula	R	47		34	22	4	6.22
Supplementary Motor Area	R	6	826	2	6	49	6.86
Cingulate Cortex	L	32		−4	17	38	5.90
Cingulate Cortex	R	8		12	29	34	5.92
Precentral gyrus	L	4	173	−36	−7	52	5.96
Intraparietal Sulcus	L	7	62	−24	−52	45	5.40
Intraparietal Sulcus	R	7	165	36	−44	43	4.36
Superior Occipital Gyrus	R	19		30	−55	34	5.72
Middle Occipital Cortex	L	18	217	−30	−77	13	7.06
Inferior Extrastriate Cortex	L	19	82	−32	−68	−7	4.50
Fusiform Gyrus	L	37		−44	−63	−12	3.85
Inferior Extrastriate Cortex	R	18	640	36	−68	0	5.85
Inferior Occipital Gyrus	R	19		30	−76	0	4.90
Fusiform Gyrus	R	37		34	−63	−9	4.35
Cerebellum	R	—		38	−54	−23	5.12
Cerebellum	L	—	1768	−30	−54	−28	6.75
Thalamus	L	—	3583	−16	−25	10	6.41
Thalamus	R	—		12	−21	12	7.47
Parahippocampal Gyrus	L	—		−16	−29	−7	3.96
Hippocampus	L	—		−22	−35	4	4.91
Hippocampus	R	—		22	−33	−2	3.92
Substantia Nigra	L	—		−12	−18	−11	4.41
Substantia Nigra	R	—		12	−18	−11	4.52
Nucleus Ruber	L	—		−8	−18	−2	6.92
Nucleus Ruber	R	—		8	−18	−2	5.89
Nucleus Subthalamicus	L	—		−12	−16	−4	6.77
Nucleus Subthalamicus	R	—		12	−16	−4	5.33
**Execution:** **No face memory > Face memory**							
Frontal Pole	L	10	57	−12	59	14	4.65
Frontal Pole	L	10	13	−14	52	−4	4.47
Frontal Pole	L	10	13	−4	50	23	4.15
Frontal Pole	R	10	23	14	55	16	4.16
Insula	R	22	11	42	−12	2	4.69
Superior Temporal Cortex	R	21	19	67	−25	5	6.46
Superior Temporal Cortex	R	22	35	59	0	4	4.48

## Results

### Behavioral data

The mean face recognition accuracy was 78.0% (SD = 10.2), and the accuracy rates for additions were not different between the two conditions (91.4%, SD = 11.5 for the Face memory condition, and 92.7%, SD = 8.7 for the No face memory condition, *t*(15) = 0.712, *p* = 0.487).

### Functional MRI data

**Figure 2:**
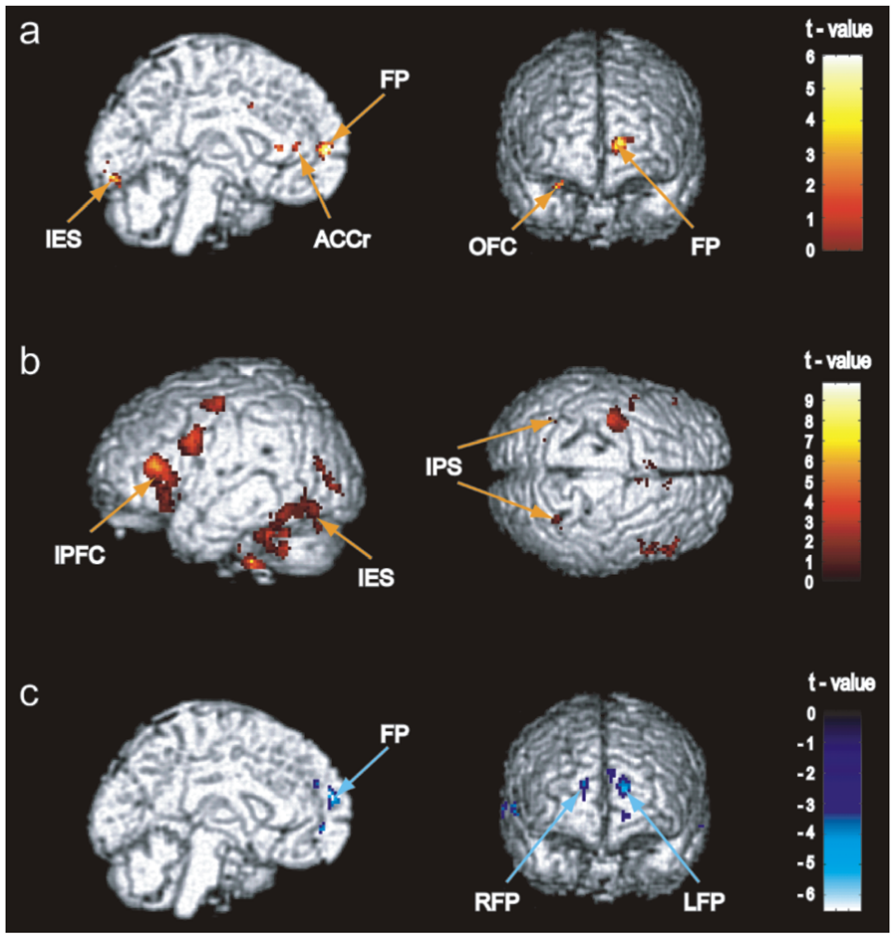
Brain activation for the task preparation and task execution periods. (a) Regions that showed activation during the preparation period in the contrast of Face Memory condition>No Face memory condition, including the left frontal pole (FP), left rostral anterior cingulate (ACCr), right orbital frontal cortex (OFC), and left inferior extrastriate (IES). No brain regions showed activation in the contrast of No Face Memory condition>Face memory condition for the preparation period. (b) Regions that showed activation during the execution period, in the contrast of Face Memory condition>No Face memory condition, including the lateral prefrontal cortex (LPFC), intraparietal sulcus (IPS), and IES. (c) Regions that showed activation during the execution period in the contrast of No Face Memory condition>Face memory condition, including bilateral FP. An uncorrected height threshold (p = 0.001) and an extent threshold (10 voxels) were used.

The brain imaging data showed that during the task preparation period, activation was greater in the left anterior MPFC, right OFC, left premotor cortex, rostral ACC, and left IES for the Face memory condition than for the No face memory condition. No brain region showed higher activation for the No face memory than for the Face memory condition during the preparation period. This is illustrated in Table 1 and Figure 2.

During the task execution period, the bilateral anterior MPFC and the lateral superior temporal cortex showed lower activation for the Face memory condition than for the No face memory condition, as shown in Table 1 and Figure 2. The brain regions that are typically associated with face working memory showed higher activation for the Face memory than for the No face memory condition. These regions included the bilateral PFC, right superior frontal gyrus, right middle frontal gyrus, left precentral gyrus, intraparietal sulcus (IPS), left fusiform gyrus, left middle occipital gyrus, and right inferior occipital gyrus.

We examined the main effect of Task period. For the Face memory condition, a contrast for Task preparation>Task execution resulted in activation in the left and right anterior medial PFC (BA 10, BA 32, BA 9), inferior orbitofrontal gyrus (BA 47), middle/superior temporal gyri (BA21, 22), inferior parietal lobe (BA 39), and posterior cingulate cortex (BA 23). This analysis identified more regions that were more active during the preparation period than the execution period than had the other analyses. Activation in the anterior MPFC is consistent with the results of the other analyses; and therefore, provided converging evidence. Activation in the middle and superior temporal gyri is related to the auditory instruction, as we obtained activation in similar regions for the No face memory condition as shown below.

For the Face memory condition, a contrast for the Task execution>Task preparation revealed activation in the face working memory network, which is very similar to the Face memory>No face memory contrast for the task execution period shown in Table 1.

For the No face memory condition, a contrast for Task preparation>Task execution resulted in activation around the auditory cortex, reflecting the auditory instruction. For the Task execution>Task preparation, participants performed calculations only, and the contrast resulted in activation in the lateral and medial frontal gyri, supplementary motor area, inferior parietal lobe, and inferior temporal and occipital cortex.

We also investigated different condition effects as a function of the task period (shown in Table S1). One was an effect in which activation was greater for the preparation than for the execution period (Preparation effect). The other was an effect in which activation was greater for the execution than for the preparation period (execution effect). We performed an interaction analysis for the preparation effect using a (Face memory for Preparation – Face memory for Execution) – (No face memory for Preparation – No face memory for Execution) contrast. This contrast identified the regions that showed the preparation effect was greater for the Face memory than for the No face memory condition, and revealed that the anterior MPFC was the only region showing this effect. When combined with the results of the other analyses, our data suggest that the anterior MPFC is activated during task preparation but deactivated during execution.

**Figure 3:**
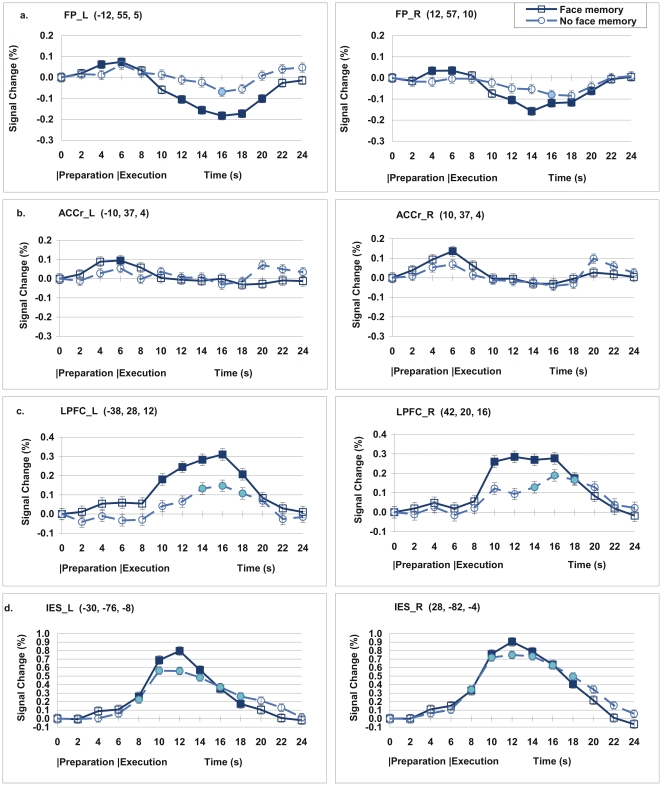
Signal change (%) across the time course. (a) Frontal pole (FP) showed activation for the Memory condition during the preparation period (6 seconds after the onset of a cue at 0 second) and deactivation during the execution period. (b) Rostral Anterior Cingulate Cortex (ACCr) exhibited activation during preparation but not during execution. (c) Lateral prefrontal cortex (LPFC) showed activation during execution. (d) Inferior extrastriate (IES) exhibited activation during execution. Inside the parentheses after each region name are the coordinates of the centre of the ROI. Error bars denote s.e.m. The filled data points indicate the points that the 99% confidence interval did not include zero. The blank data points indicate those points that the 99% confidence interval included zero. The central coordinates for each ROI is shown inside the parentheses.

**Figure 4:**
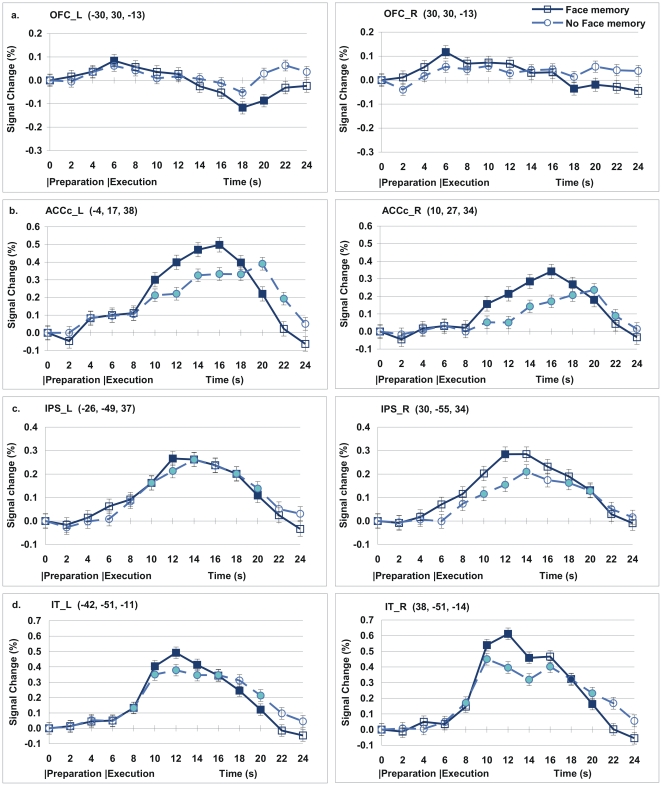
Signal change (%) across the time course. (a) Orbitofrontal cortex (OFC) showed activation during preparation and deactivation during execution. (b) Left Caudal Anterior Cingulate Cortex (ACCc) exhibited activation during preparation and execution. (c) Intraparietal sulcus (IPS) showed activation during execution. (d) Inferior Temporal cortex (IT) showed activation during execution. Inside the parentheses after each region name are the coordinates of the centre of the ROI. Error bars denote s.e.m. The filled data points indicate the points that the 99% confidence interval did not include zero. The blank data points indicate those points that the 99% confidence interval included zero. The central coordinates for each ROI is shown inside the parentheses.

We performed the other interaction analysis with the contrast (Face memory during Execution – Face memory during preparation) – (No face memory during execution – No face memory during preparation). This contrast identified the areas in which the execution effect was greater for the Face memory than for the No face memory condition. These were the regions associated with face working memory. The results of this analysis implicated exactly the regions, including lateral PFC, SMA, Inferior parietal lobe, and inferior temporal/occipital regions. These are very similar to the regions in the contrast for the Face memory>No face memory during task execution shown in Table 1. These results are shown in Table S2. Percent signal change (psc) data across the time course are shown in Figure 3 and 4. When participants formed the memory task set during the preparation period, the anterior MPFC showed activation during task preparation and deactivation during task execution compared to its own baseline. Also, the bilateral OFC, and the bilateral rostral ACC exhibited activation during task preparation, but showed neither activation nor deactivation during task execution. During the task execution period, the bilateral PFC, the caudal ACC (ACCc), and the posterior regions including the bilateral IPS, bilateral inferior temporal (IT), and bilateral IES, showed increased activation regardless of memory task set. Among the regions that are typically related to face working memory, the lateral PFC showed activation for the Face memory condition, but not for the No face memory condition. The other posterior regions including the IPS, IT, and IES all showed activations for both conditions.

## Discussion

The results of the analyses, including the difference between the Face memory and No face memory conditions, between task preparation and execution, the interactions, and the percent signal change, all converge into the following points. During the preparation period, the anterior MPFC and IES showed activation. During the execution period, the regions that are typically associated with face working memory showed activation, including the lateral PFC, IPS, IT, and IES [78], [79]. However, during the execution period, the anterior MPFC and the superior temporal cortex showed deactivation.

In the present study, the anterior MPFC exhibited activation during the task preparation period but deactivation during the task execution period within a single trial. The results based on the comparison between the Face memory and No face memory condition and the percent signal change data converged into the same results. The activation during the preparation period is consistent with previous findings that the anterior MPFC plays a role in initiation and management of task sets [21], [30], [31], [34], [35].

Deactivation in the anterior MPFC during the execution period would be viewed as TID in the prefrontal regions. In previous research, it was shown that reduction of activation could be due to allocation of attentional resources within posterior regions, such as the occipital lobe [67]–[69], somatosensory cortex [80]–[82], and temporoparietal junction [70]–[72]. Prior research has also shown that the effect of allocation of attentional resources could be global, including between different hemispheres [73], and TID is not due to local blood stealing but rather to dynamic changes of global neural activity [47], [73]. Therefore, TID would reflect dynamic shifts of attentional resources in the whole brain including the prefrontal regions. When neural activities in some regions increase, those in other regions might decrease as a function of resource demands.

In the present study, deactivation in the anterior MPFC during the execution period could be the results of two factors. One is that the anterior MPFC is involved in task set formation, but not in execution of face working memory. The other is that activation and deactivation are affected by allocation of attentional resources. In our task, the anterior MPFC was active during the task preparation period because it plays an important role in task preparation, whereas it was deactivated during task execution because (1) the anterior MPFC is not involved in execution of face working memory, (2) our task was relatively simple and did not require maintenance of task set during the execution period, and (3) resource demands in the other regions of the brain increased to perform the face working memory task. The brain is a limited capacity information processing system; and therefore, as the processing demands in the other frontal and posterior regions increased during the execution period, the attentional resources were dynamically shifted to those regions, resulting in deactivation in the anterior MPFC. If the task required maintenance of complex task sets or continuous switching between task sets, the anterior MPFC might have shown sustained activation during execution [21], [31], [32], [34].

In this study, we measured activation and deactivation in two ways. One is based on the voxelwise analysis, in which results showed that the level of activation in the anterior MPFC was higher for the Face memory condition than for the No Face memory condition during the preparation period (Table 1 and Figure 2). However, the level of activation in the anterior MPFC was lower for the Face memory condition than for the No face memory condition during the task execution period. The other analysis was based on the percent signal change (psc), in which the level of activation for each ROI was measured in comparison with its own baseline at the time point zero, as shown in Figures 3 & 4. The psc for the anterior MPFC increased during preparation, and then decreased during execution within a single trial.

One might question whether or not the time point zero is a valid baseline. If the DMN is active during the ITI period, and therefore, the anterior MPFC is already active at the point of instruction, then the decreased level of psc during execution might not be reflecting deactivation, but instead reflecting the fact that the level of activation is going back to the true baseline. However, if this is the case, then we should observe the same pattern of decrease in psc for the No face memory condition during the task execution period because participants did not know which condition would be their next trial during the ITI period. However, the psc for the No face memory condition stayed basically flat around the zero percent for the prefrontal regions, indicating that the level of activation at the time point zero was not significantly influenced by activities during the ITI period; and therefore, the time point zero provided a valid estimate of the baseline. Therefore, the results of these two analyses converge to support the claim that the anterior MPFC showed activation during task preparation and deactivation during task execution.

The inferior extrastriate cortex also showed activation during the preparation period, consistent with previous findings in the effects of top-down attention on the posterior regions [67], [83]–[85]. In our study, when participants were expecting face stimuli, they formed a task set including the IES. This activation in the IES continued into the execution period because of the role IES plays in visual information processing.

Our results might also shed some light on the mechanism of DMN, which shows deactivation during some cognitive tasks compared to a resting baseline [44]–[51]. This deactivation has been explained in terms of activities during the rest period, including monitoring the external environment [30] and processing of internal states or mind wandering [52], [53]. One of the interesting differences between the previous studies on the DMN and our study is that in the previous studies, deactivation in the DMN has been observed in the comparison between the rest and task conditions in different blocks, whereas, in our study, the task preparation and execution periods occurred within a single trial. Therefore, our results suggest that deactivation could also be related to dynamic temporal shifts of attentional resources among the brain regions. In other words, deactivation in the anterior MPFC could be due not only to activities during the resting condition, but also to reduction of activity for the task condition because of heightened activities in other brain regions. This is consistent with the findings of previous studies that have pointed out the negative relations between the DMN and executive network [52], [54]–[58], as well as the DMN and task demands [59].

Among the other brain regions that are typically included in the DMN, such as the posterior cingulate cortex (PCC), inferior parietal lobe (IPL), lateral temporal cortex (LTC), and hippocampal formation (HF) [45], the lateral superior temporal cortex showed deactivation during the task execution period, while the other regions did not show deactivation in our study. The results seem to suggest that even though the brain regions in the DMN tend to be related to each other, they might not show the same pattern of activity in some cases, depending on their roles and resource demands in a given task. In our study, the bilateral rostral ACC showed activation during preparation. However, during the execution period, the ACC did not show deactivation, because they play an important role in task monitoring [86], [87]. Among other regions related to DMN, the lateral superior temporal regions showed deactivation, probably because these regions do not have significant roles in the face working memory task. Thus, our results were not inconsistent with the previous findings regarding the DMN. Our results simply suggest that not all the DMN regions have to show the same pattern of activation and deactivation in all kinds of cognitive tasks. Rather, activation and deactivation of those regions are influenced by a number of factors including their involvement in a given phase of a task, overall task demands, and the distribution of processing resources among the brain regions.

In conclusion, our results showed that the anterior MPFC exhibited activation during task preparation but deactivation during task execution within a single trial, suggesting that the temporal dynamics of activity in the anterior MPFC are affected by a number of factors. First, the anterior MPFC has a role in task preparation. Second, the anterior MPFC shows deactivation when it is not involved in task execution and when activity in the other brain regions increases. In the present study, the other regions in the DMN did not show the same pattern of deactivation as the anterior MPFC during task execution. Therefore, our results suggest that the activation and deactivation in the regions of the DMN might depend on the roles they play in a given task and resource demands of the task. Future research will be needed to determine the conditions under which patterns of activation or deactivation are similar across regions of the DMN and conditions under which the patterns differ.

## Supporting Information

Table S1 Areas of activation for the Face Memory and No Face memory conditions between the Preparation and Execution phases.
(DOC)

Table S2 Areas of activation for the interaction analyses.
(DOC)

## References

[1] Lepage M, Ghaffar O, Nyberg L, Tulving W (2000). Prefrontal cortex and episodic memory retrieval mode.. Proc Natl Acad Sci USA.

[2] MacLeod AK, Buckner RL, Miezin FM, Petersen SE, Raichle ME (1998). Right anterior prefrontal cortex activation during semantic and working memory.. Neuroimage.

[3] Nolde SF, Johnson MK, D'Esposito M (1998). Left prefrontal activation during episodic remembering: an event-related fMRI study.. Neuroreport.

[4] Nyberg L, McIntosh AR, Cabeza R, Habib R, Houles S (1996). General and specific brain regions involved in encoding and retrieval of events: what, where, and when.. Proc Natl Acad Sci USA.

[5] Ranganath C, Johnson MK, D'Esposito M (2003). Prefrontal activity associated with working memory and episodic long-term memory.. Neuropsychologia.

[6] Rugg MD, Wilding EL (2000). Retrieval processing and episodic memory.. Trends Cogn Sci.

[7] Tulving E, Kapur S, Craik FIM, Moscovitch M, Houle S (1994). Hemispheric encoding/retrieval asymmetry in episodic memory: Positron emission tomography findings.. Proc Natl Acad Sci USA.

[8] Velanova K, Jacoby LL, Wheeler ME, McAvoy MP, Petersen SE (2003). Functional-anatomic correlates of sustained and transient processing components engaged during controlled retrieval.. J Neurosci.

[9] Burgess PW, Quayle A, Frith CD (2001). Brain regions involved in prospective memory as determined by positron emission tomography.. Neuropsychologia.

[10] Okuda J, Fujii T, Yamadori A, Kawashima R, Tsukiura T (1998). Participation of the prefrontal cortices in prospective memory: evidence from a PET study in humans.. Neurosci Letters.

[11] Simons JS, Scholvinck M, Gilbert SJ, Frith CD, Burgess PW (2006). Differential components of prospective memory? Evidence from fMRI.. Neuropsychologia.

[12] Owen AM, McMillan KM, Laird AR, Bullmore E (2005). N-back working memory paradigm: A meta-analysis of normative functional neuroimaging studies.. Hum Brain Mapp.

[13] de Zubicaray GI, Zelaya FO, Andrew C, Williams SCR, Bullmore ET (2000). Cerebral regions associated with verbal response initiation, suppression and strategy use.. Neuropsychologia.

[14] Fan J, Flombaum JI, McCandltss BD, Thomas KM, Posner MI (2003). Cognitive and brain consequences of conflict.. Neuroimage.

[15] Pollmann S (2001). Switching between dimensions, locations, and responses: the role of the left frontopolar cortex.. Neuroimage.

[16] Pollmann S, Weidner R, Muller HJ, von Cramon DY (2000). A fronto-posterior network involved in visual dimension changes.. J Cogn Neurosci.

[17] Baker SC, Rogers RD, Owen AM, Frith CD, Dolan RJ (1996). Neural systems engaged by planning: a PET study of the Tower of London task.. Neuropsychologia.

[18] Kroger JK, Sabb FW, Fales CL, Bookheimer SY, Cohen MS (2002). Recruitment of anterior dorsolateral prefrontal cortex in human reasoning: a parametric study of relational complexity.. Cereb Cortex.

[19] Braver TS, Bongiolatti SR (2002). The role of frontpolar cortex in subgoal processing during working memory.. Neuroimage.

[20] Braver TS, Reynolds JR, Donaldson DI (2003). Neural mechanisms of transient and sustained cognitive control during task switching.. Neuron.

[21] Koechlin E, Basso G, Peitrini P, Panzer S, Grasby PM (1999). The role of the anterior prefrontal cortex in human cognition.. Nature.

[22] Koechlin E, Hyafil A (2007). Anterior prefrontal function and the limits of human decision making.. Science.

[23] Ramnani N, Owen AM (2004). Anterior prefrontal cortex: Insights into function from anatomy and neuroimaging.. Nat Rev Neurosci.

[24] Bunge SA (2004). How we use rules to select actions: A review of evidence from cognitive neuroscience.. Cogn Affect Behav Neurosci.

[25] Koechlin E, Danek A, Burnod Y, Grafman J (2002). Medial prefrontal and subcortical mechanisms underlying the acquisition of motor and cognitive action sequences in humans.. Neuron.

[26] Strange BA, Henson RN, Friston KJ, Dolan RJ (2001). Anterior prefrontal cortex mediates rule learning in humans.. Cereb Cortex.

[27] Yoshida W, Ishii S (2006). Resolution of uncertainty in prefrontal cortex.. Neuron.

[28] Christoff K, Gabrieli JDE (2000). The frontopolar cortex and human cognition: evidence for a rostrocaudal hierarchical organization within the human prefrontal cortex.. Psychobiology.

[29] Christoff K, Ream JM, Geddes JPT, Gabrieli JDE (2003). Evaluating self-generated information: anterior prefrontal contribution to human cognition.. Behav Neurosci.

[30] Burgess PW, Dumontheil I, Gilbert SJ (2007). The gateway hypothesis of rostral prefrontal cortex (area 10) function.. Trends Cogn Sci.

[31] Dosenbach NU, Visscher KM, Palmer ED, Miezin FM, Wenger KK (2006). A core system for the implementation of task sets.. Neuron.

[32] Haynes J-D, Sakai K, Rees G, Gilbert S, Frith C (2007). Reading hidden intentions in the human brain.. Curr Biol.

[33] Rowe JB, Sakai K, Lund TE, Ramsoy T, Christensen MS (2007). Is the prefrontal cortex necessary for establishing cognitive sets?. J Neurosci.

[34] Sakai K, Passingham RE (2003). Prefrontal interactions reflect future task operations.. Nat Neurosci.

[35] Sakai K, Passingham RE (2006). Prefrontal set activity predicts rule-specific neural processing during subsequent cognitive performance.. J Neurosci.

[36] Daw ND, O'Doherty JP, Dayan P, Seymour B, Dolan RJ (2006). Cortical substrates for exploratory decisions in humans.. Nature.

[37] Soon CS, Brass M, Heinze H-J, Haynes J-D (2008). Unconscious determinants of free decisions in the human brain.. Nat Neurosci.

[38] Rameson LT, Satpute AB, Lieberman MD (2010). The neural correlates of implicit and explicit self-relevant processing.. Neuroimage.

[39] Zysset S, Huber O, Samson A, Ferstl EC, von Cramon DY (2002). Functional specialization within the anterior medial prefrontal cortex: a functional magnetic resonance imaging study with human subjects.. Neurosci Let.

[40] Bengtsson SL, Haynes J-D, Sakai K, Buckley MJ, Passingham RE (2009). The representation of abstract task rules in the human prefrontal cortex.. Cereb Cortex.

[41] Burgess PW, Gilbert SJ, Dumontheli I (2007). Functional and localization within rostral prefrontal cortex (area 10).. Phil Trans R Soc B.

[42] Gilbert SJ, Spengler S, Simons JSS, Steele JD, Lawrie SM (2006). Functional specialization within rostral prefrontal cortex (area 10): a meta-analysis.. J Cogn Neurosci.

[43] Burgess PW, Veitch E, de Lacy Costello A, Shallice T (2000). The cognitive and neuroanatomical correlates of multitasking.. Neuropsychologia.

[44] Binder JR, Frost JA, Hammeke TA, Bellgowan PSF, Rao SM (1999). Conceptual processing during the conscious resting state: a functional MRI study.. J Cogn Neurosci.

[45] Buckner RL, Andrews-Hanna JR, Schacter DL (2008). The brain's default network: anatomy, function, and relevance to disease.. Ann New York Acad Sci.

[46] Gusnard DA, Raichle ME (2001). Searching for a baseline: Functional imaging and the resting human brain.. Nat Rev Neurosci.

[47] Raichle ME, MacLeod AM, Snyder AZ, Powers WJ, Gusnard DA (2001). A default mode of brain function.. Proc Natl Acad Sci USA.

[48] Mazoyer B, Zago L, Mellet E, Bricogne S, Etard O (2001). Cortical networks for working memory and executive functions sustain the conscious resting state in man.. Brain Res Bull.

[49] McKiernan KA, Kaufman JN, Kucera-Thompson J, Binder JR (2003). A parametric manipulation of factors affecting task-induced deactivation in functional Neuroimaging.. J Cogn Neurosci.

[50] Shulman GL, Fiez JA, Corbetta M, Buckner RL, Miezin FM (1997). Common blood flow changes across visual tasks: II. Decreases in cerebral cortex.. J Cogn Neurosci.

[51] Sridharan D, Levitin DJ, Menon V (2008). A critical role for the right fronto-insular cortex in switching between central-executive and default-mode networks.. Proc Natl Acad Sci USA.

[52] Christoff K, Gordon AM, Smallwood J, Smith R, Schooler JW (2009). Experience sampling during fMRI reveals default network and executive system contributions to mind wandering.. Proc Natl Acad Sci USA.

[53] Mason MF, Norton MI, Van Horn JD, Wegner DM, Grafton ST (2007). Wandering minds: the default network and stimulus-independent thought.. Science.

[54] Fox MD, Snyder AZ, Vincent JL, Corbetta M, Van Essen DC (2005). The human brain is intrinsically organized into dynamic, anticorrelated functional networks.. Proc Natl Acad Sci USA.

[55] Grecius MD, Krasnow B, Reiss AL, Menon V (2003). Functional connectivity in the resting brain: A network analysis of the default mode hypothesis.. Proc Natl Acad Sci USA.

[56] Hampson M, Driesen NR, Skudlarski P, Gore JC, Constable RT (2006). Brain connectivity related to working memory performance.. J Neurosci.

[57] Tomasi D, Ernst T, Caparelli EC, Chang L (2006). Common deactivation patterns during working memory and visual attention tasks: An intrasubject fMRI study at 4 Tesla.. Hum Brain Map.

[58] Weissman DH, Roberts KC, Visscher KM, Woldorff MG (2006). The neural basis of momentary lapses in attention.. Nat Neurosci.

[59] Mayer JS, Roebroeck A, Maurer K, Linden DEJ (2010). Specialization in the default mode: Task-induced brain deactivations dissociate between visual working memory and attention.. Hum Brain Mapp.

[60] Corbetta M, Shulman GL (2002). Control of goal-directed and stimulus driven attention in the brain.. Nat Rev Neurosci.

[61] Corbetta M, Patel G, Shulman GL (2008). The reorienting system of the human brain: From environment to theory of mind.. Neuron.

[62] Hopfinger JB, Buonocore MH, Mangun GR (2000). The neural mechanisms of top-down attentional control.. Nat Neurosci.

[63] Kastner S, Ungerleider LG (2000). Mechanisms of visual attention in the human cortex.. Annual Rev Neurosci.

[64] O'Craven KM, Downing PE, Kanwisher N (1999). fMRI evidence for objects as the units of attentional selection.. Nature.

[65] Serences J, Yantis S, Culberson A, Awh E (2004). Preparatory activity in visual cortex indexes distractor suppression during covert spatial orienting.. J Neurophysiol.

[66] Kastner S, De Weerd P, Desimone R, Ungerleider LG (1998). Mechanisms of directed attention in the human extrastriate cortex as revealed by functional MRI.. Science.

[67] Kastner S, Pinsk MA, De Weerd P, Desimone R, Ungerleider LG (1999). Increased activity in human visual cortex during directed attention in the absence of visual stimulation.. Neuron.

[68] Shmuel A, Yacoub E, Pfeuffer J, Van de Moortele P-F, Adriany G (2002). Sustained negative BOLD, blood flow and oxygen consumption response and its coupling to the positive response in the human brain.. Neuron.

[69] Tootell RBH, Hadjikhani N, Hall EK, Marrett S, Vanduffel W (1998). The retinotopy of visual spatial attention.. Neuron.

[70] Matsuyoshi D, Ikeda T, Sawamoto N, Kakigi R, Fukuyama H (2010). Task-irrelevant memory load induces inattentional blindness without temporo-parietal suppression.. Neuropsychologia.

[71] Shulman GL, McAvoy MP, Cowan MC, Astafiev SV, Tansy AP (2003). Quantitative analysis of attention and detection signals during visual search.. J Neurophysiol.

[72] Todd JJ, Fougnie D, Marois R (2005). Visual-short term memory load suppresses temporo-parietal junction activity and induces inattentional blindness.. Psychol Sci.

[73] Smith AT, Williams AL, Singh KD (2004). Negative BOLD in the visual cortex: Evidence against blood stealing.. Hum Brain Map.

[74] Minear M, Park DC (2004). A lifespan database of adult facial stimuli.. Behav Res Methods Instrum Comput.

[75] Minamoto T, Osaka M, Osaka N (2010). Individual Differences in Working Memory Capacity and Distractor Processing: Possible Contribution of Top-Down Inhibitory Control.. Brain Research.

[76] Koshino H, Kana RK, Keller TA, Cherkassky VL, Minshew NJ (2008). fMRI investigation of working memory for faces in autism: visual coding and underconnectivity with frontal areas.. Cereb Cortex.

[77] Brett M, Anton JL, Valabregue R, Poline JB (2002). Region of interest analysis using an SPM toolbox.. the 8th International Conference on Functional Mapping of the Human Brain.

[78] Haxby JV, Horwitz B, Ungerleider LG, Maisog JM, Pietrini P (1994). The functional organization of human extrastriate cortex: A PET-rCBF study of selective attention to faces and locations.. J Neurosci.

[79] Haxby JV, Ungerleider LG, Horwitz B, Maisog JM, Rapoport SI (1996). Face encoding and recognition in the human brain.. Proc Natl Acad Sci USA.

[80] Drevets WC, Burton H, Videen TO, Snyder AZ, Simpson JR (1995). Blood flow changes in human somatosensory cortex during anticipated stimulation.. Nature.

[81] Kastrup A, Baudewig J, Schnaudigel S, Huonker R, Becker L (2008). Behavioral correlates of negative BOLD signal changes in the primary somatosensory cortex.. Neuroimage.

[82] Laurienti PJ, Burdette JH, Wallace MT, Yen Y-F, Field AS (2002). Deactivation of sensory-specific cortex by cross-modal stimuli.. J Cogn Neurosci.

[83] Gazzaley A, Cooney JW, McEvoy K, Knight RT, D'Esposito M (2005). Top-down enhancement and suppression of the magnitude and speed of neural activity.. J Cogn Neurosci.

[84] Miller BT, D'Esposito M (2005). Search for “the top” in top-down control.. Neuron.

[85] Pessoa L, Kastner S, Ungerleider LG (2003). Neuroimaging studies of attention: From modulation of sensory processing to top-down control.. J Neurosci.

[86] Botvinick M, Nystrom LE, Fissell K, Carter CS, Cohen JD (1999). Conflict monitoring versus selection-for-action in anterior cingulate cortex.. Nature.

[87] Bush G, Luu P, Posner MI (2000). Cognitive and emotional influences in anterior cingulate cortex.. Trends Cogn Sci.

