# Supplementary material for: Anterior Medial Prefrontal Cortex Exhibits Activation during Task Preparation but Deactivation during Task Execution
Source: PLoS One. 2011 Aug 1;6(8):e22909. doi: 10.1371/journal.pone.0022909 (PMC3148238; doi:10.1371/journal.pone.0022909)
Supplement: Table S1 — Areas of activation for the Face Memory and No Face memory conditions between the Preparation and Execution phases. (DOC) [file pone.0022909.s001.doc]

Table S1. Areas of activation for the Face Memory and No Face memory conditions between the Preparation and Execution phases.

| Region | L/R | BA | Cluster size | x | y | z | T-score |
| --- | --- | --- | --- | --- | --- | --- | --- |
| Memory: Preparation > Execution | |  |  |  |  |  |  |
| Frontal Pole | L | 10 | 1348 | -8 | 54 | 6 | 7.73 |
| Medial superior frontal gyrus | L | 32 |  | -6 | 48 | 30 | 6.11 |
| Superior frontal gyrus | L | 9 |  | -12 | 56 | 28 | 5.98 |
| Superior frontal gyrus | R | 9 | 30 | 20 | 34 | 48 | 4.58 |
| Inferior orbitofrontal gyrus | L | 47 | 66 | -46 | 36 | -12 | 5.2 |
| Middle temporal gyrus | R | 22 | 2846 | 66 | -20 | -4 | 9.52 |
| Superior temporal gyrus | R | 48 |  | 58 | -6 | 0 | 7.12 |
| Superior temporal gyrus | R | 22 |  | 60 | -8 | -8 | 6.8 |
| Middle temporal gyrus | L | 48 | 2074 | -54 | -12 | -6 | 8.65 |
| Middle temporal gyrus | L | 21 |  | -54 | -4 | -14 | 6.36 |
| Superior temporal gyrus | L | 42 |  | -60 | -38 | 16 | 6.34 |
| Posterior cingulate cortex | L | 23 | 374 | -8 | -44 | 30 | 5.35 |
| Precuneus | L | 23 |  | -8 | -60 | 20 | 4.89 |
| Middle temporal gyrus | L | 39 | 111 | -42 | -66 | 20 | 5.91 |
| Middle temporal gyrus | L | 39 |  | -46 | -60 | 24 | 5.63 |
| Cerebelum | R | n/a | 24 | 22 | -78 | -42 | 6.76 |
|  |  |  |  |  |  |  |  |
|  |  |  |  |  |  |  |  |
| Memory: Execution > Preparation | |  |  |  |  |  |  |
| Supplementary motor area | R | 32 | 1085 | 2 | 12 | 50 | 8.35 |
| Medial superior frontal gyrus | L | 32 |  | -8 | 22 | 42 | 8.07 |
| Supplementary motor area | L | 6 |  | -10 | 2 | 54 | 7.96 |
| Precentral gyrus | L | 6 | 2028 | -30 | -14 | 50 | 7.96 |
| Precentral gyrus | L | 44 |  | -44 | 4 | 34 | 7.8 |
| Inferior frontal gyrus | L | 48 |  | -40 | 10 | 24 | 7.27 |
| Middle frontal gyrus | R | 6 | 80 | 32 | 0 | 54 | 5.35 |
| Inferior frontal gyrus | R | 48 | 1073 | 46 | 14 | 24 | 7.25 |
| Insula | R | 48 |  | 34 | 18 | 10 | 6.15 |
|  | R | 48 |  | 38 | -2 | 22 | 4.72 |
| Inferior frontal gyrus | R | 45 | 212 | 44 | 32 | 16 | 6.14 |
| Inferior parietal lobe | L | 40 | 16 | -50 | -32 | 40 | 4.01 |
| Inferior parietal lobe | L | 40 |  | -44 | -38 | 44 | 3.84 |
| Inferior parietal lobe | R | 40 | 463 | 32 | -50 | 46 | 6.14 |
| Middle occipital gyrus | R | 19 |  | 30 | -64 | 36 | 5.55 |
| Cuneus | R | 7 |  | 18 | -64 | 40 | 4.42 |
| Inferior temporal gyrus | R | 37 | 1664 | 44 | -56 | -12 | 8.07 |
| Lingual gyrus | R | 18 |  | 26 | -84 | -4 | 6.8 |
| Fusiform gyrus | R | 37 |  | 40 | -48 | -22 | 6.6 |
| Inferior occipital gyrus | L | 19 | 2479 | -42 | -76 | -8 | 8.66 |
| Inferior occipital gyrus | L | 18 |  | -26 | -88 | -2 | 8.62 |
| Inferior occipital gyrus | L | 37 |  | -42 | -58 | -10 | 6.96 |
|  | R | n/a | 290 | 4 | -32 | -6 | 7.15 |
|  | L | n/a |  | -8 | -26 | -10 | 5.28 |
| Thalamus | L | n/a | 107 | -8 | -12 | 6 | 5.5 |
|  | R | n/a | 33 | 4 | 6 | 26 | 5.24 |
| Thalamus | R | n/a | 102 | 8 | -16 | 8 | 5.02 |
| Pallidum | L | n/a | 174 | -18 | 8 | 0 | 4.86 |
|  | L | n/a |  | -20 | 2 | 14 | 4.8 |
|  | L | n/a |  | -20 | -8 | 20 | 3.92 |
|  | L | n/a | 63 | -20 | -30 | 12 | 4.49 |
|  | L | n/a | 12 | -30 | -54 | -38 | 4.25 |

| Region | L/R | BA | Cluster size | x | y | z | T-score |
| --- | --- | --- | --- | --- | --- | --- | --- |
| No Memory: Preparation > Execution | |  |  |  |  |  |  |
| Superior temporal gyrus | R | 21 | 1283 | 58 | -32 | 6 | 6.01 |
| Superior temporal gyrus | R | 21 |  | 68 | -22 | 0 | 5.71 |
| Superior temporal gyrus | R | 48 |  | 56 | -12 | -2 | 5.4 |
| Middle temporal gyrus | L | 22 | 1224 | -56 | -10 | -6 | 5.82 |
| Middle temporal gyrus | L | 21 |  | -52 | -24 | -4 | 5.67 |
| Superior temporal gyrus | L | 22 |  | -60 | -24 | 4 | 5.19 |
| Cerebelum | R | n/a | 93 | 4 | -66 | -4 | 4.62 |
| Cerebelum | L | 18 |  | -12 | -64 | -12 | 4.22 |
| Cuneus | L | n/a | 21 | -4 | -72 | 26 | 4.13 |
|  |  |  |  |  |  |  |  |
|  |  |  |  |  |  |  |  |
| No Memory: Execution > Preparation | |  |  |  |  |  |  |
| Inferior frontal gyrus | L | 48 | 1283 | -34 | 12 | 26 | 10.68 |
| Precentral gyrus | L | 6 |  | -44 | 2 | 34 | 8.31 |
| Precentral gyrus | L | 6 |  | -28 | -10 | 48 | 7.25 |
| Medial superior frontal gyrus | L | 32 | 438 | -8 | 20 | 44 | 6.57 |
| Supplementary motor area | R | 32 |  | 8 | 12 | 48 | 6.45 |
| Supplementary motor area | L | 6 |  | -10 | 0 | 56 | 5.74 |
|  | R | 48 | 557 | 42 | 14 | 22 | 6.21 |
| Precentral gyrus | R | 44 |  | 48 | 8 | 34 | 5.4 |
| Insula | L | 48 | 65 | -32 | 22 | 6 | 5.1 |
| Middle frontal gyrus | R | 6 | 23 | 32 | -2 | 54 | 4.41 |
|  | R | 7 | 384 | 24 | -50 | 48 | 8.02 |
| Inferior parietal lobe | R | 40 |  | 32 | -50 | 46 | 6.96 |
| Middle occipital gyrus | R | 19 |  | 30 | -64 | 36 | 5.37 |
| Inferior occipital gyrus | L | 19 | 1825 | -42 | -76 | -8 | 11.07 |
| Inferior occipital gyrus | L | 19 |  | -32 | -86 | -6 | 8.83 |
| Inferior occipital gyrus | L | 18 |  | -24 | -90 | -4 | 8.25 |
| Lingual gyrus | R | 18 | 1086 | 20 | -88 | -10 | 7.26 |
| Inferior occipital gyrus | R | 19 |  | 30 | -82 | -10 | 7.09 |
| Fusiform gyrus | R | 37 |  | 40 | -50 | -20 | 6.56 |
| Caudate | L | 0 | 12 | -14 | 24 | 2 | 4.7 |
|  | L | 0 | 12 | -22 | 8 | 34 | 4.43 |
|  | L | 0 | 14 | -18 | 24 | 20 | 4.22 |
