# Supplementary material for: Anterior Medial Prefrontal Cortex Exhibits Activation during Task Preparation but Deactivation during Task Execution
Source: PLoS One. 2011 Aug 1;6(8):e22909. doi: 10.1371/journal.pone.0022909 (PMC3148238; doi:10.1371/journal.pone.0022909)
Supplement: Table S2 — Areas of activation for the interaction analyses. (DOC) [file pone.0022909.s002.doc]

Table S2. Areas of activation for the interaction analyses.

| Region | L/R | BA | Cluster size | x | y | z | T-score |
| --- | --- | --- | --- | --- | --- | --- | --- |
| (Mem Prep – Mem Exe) - (No Mem Prep - No Mem Exe) | | | |  |  |  |  |
| Orbitofrontal cortex | L | 11 | 180 | -18 | 56 | 0 | 6.1 |
| Frontal pole | L | 10 |  | -10 | 62 | 12 | 5.98 |
| Frontal pole | R | 10 | 41 | 12 | 58 | 12 | 5.3 |
|  |  |  |  |  |  |  |  |
| (Mem Exe - Mem Prep - (No Mem Exe - No Mem Prep) | | | |  |  |  |  |
| Medial superior frontal gyrus | R | 32 | 494 | 6 | 30 | 40 | 7.73 |
| Middle cungulate cortex | L | 32 |  | -6 | 22 | 40 | 6.24 |
| Supplementary motor area | R | 6 |  | 4 | 0 | 56 | 4.7 |
| Precentral gyrus | L | 6 | 15 | -30 | -2 | 50 | 4.3 |
| Inferior frontal gyrus | L | 45 | 145 | -40 | 22 | 6 | 6.18 |
| Insula | L | 47 |  | -34 | 26 | 2 | 4.83 |
| Inferior frontal gyrus | L | 47 |  | -36 | 34 | 2 | 4.68 |
| Insula | R | 48 | 85 | 36 | 20 | 8 | 5.37 |
| Inferior frontal gyrus | L | 48 | 222 | -40 | 6 | 24 | 5.21 |
|  | L | 48 |  | -36 | 14 | 22 | 4.65 |
|  | R | 48 | 148 | 40 | 12 | 20 | 4.73 |
| Inferior frontal gyrus | R | 44 |  | 48 | 12 | 24 | 4.41 |
| Inferior frontal gyrus | R | 45 | 88 | 52 | 30 | 20 | 4.99 |
| Pallidum | L | 48 | 17 | -14 | 0 | -4 | 4.22 |
| Anterior cingulate cortex | L | n/a | 14 | -6 | 4 | 30 | 4.12 |
|  | R | n/a |  | 0 | 8 | 22 | 3.81 |
| Angular gyrus | L | 7 | 23 | -32 | -58 | 36 | 5.82 |
| Middle temporal gyrus | R | 42 | 16 | 58 | -42 | 12 | 4.19 |
|  | L | n/a | 11 | -4 | -24 | -20 | 4.09 |
|  | L | n/a | 15 | -22 | -40 | -32 | 4.87 |
| Fusiform gyrus | L | 37 | 16 | -38 | -44 | -24 | 4.22 |
| Fusiform gyrus | R | 37 | 18 | 40 | -54 | -14 | 4.01 |
| Inferior occipital gyrus | R | 37 | 75 | 36 | -66 | -8 | 5.07 |
| Middle occipital gyrus | L | 19 | 27 | -30 | -76 | 18 | 4.74 |
| Thalamus | L | n/a | 25 | -18 | -28 | 2 | 4.48 |
| Thalamus | L | n/a | 361 | -12 | -8 | 4 | 6.76 |
|  | L | n/a |  | -10 | -18 | -6 | 5.05 |
|  | R | n/a |  | 10 | -20 | -10 | 4.89 |
| Cerebelum | R | n/a | 17 | 32 | -60 | -30 | 4.36 |
|  | L | n/a | 10 | -8 | -40 | -26 | 4.3 |
|  | R | n/a | 36 | 20 | -2 | 12 | 4.26 |
